# Delayed diagnosis of X-linked hypophosphatemia in the absence of family history: a global unmet need

**DOI:** 10.1093/jbmrpl/ziaf158

**Published:** 2025-10-07

**Authors:** Suma Uday, M Zulf Mughal, Hamilton Cassinelli, Pablo Florenzano, Erik A Imel, Jill Simmons, Leanne M Ward, Shubham Tewari, Osman Ciğeroğlu, Zhiyi Li, Ben Johnson, Kerry Sandilands, Thomas O Carpenter

**Affiliations:** Birmingham Women’s and Children’s NHS Foundation Trust, Department of Endocrinology, Birmingham, B4 6NH, United Kingdom; Department of Metabolism and Systems Science, University of Birmingham, Birmingham, B15 2TT, United Kingdom; Department of Pediatric Endocrinology, Al Jalila Children’s Specialty Hospital, Al Jaddaf, Dubai, 7662, UAE; Division de Endocrinologia, Hospital de Niños, C1425 Buenos Aires, Argentina; Departamento Endocrinología, Escuela de Medicina, Pontificia Universidad Católica de Chile, 8331150 Santiago, Chile; Departments of Medicine and Pediatrics, Indiana University School of Medicine, Indianapolis, Indiana 46202-3082, United States; Department of Pediatrics, Division of Endocrinology and Diabetes, Vanderbilt University School of Medicine, Vanderbilt University, Nashville, Tennessee 37232, United States; Department of Pediatrics, Faculty of Medicine, University of Ottawa, Ottawa, Ontario, K1H 8M5, Canada; KMK Consulting Inc., Morristown, New Jersey 07950, United States; Ultragenyx Pharmaceutical Inc., Novato, California 94949, United States; Kyowa Kirin, Princeton, New Jersey 08540, United States; Kyowa Kirin, Marlow, Buckinghamshire, SL7 1HZ, United Kingdom; Kyowa Kirin, Galashiels, Scottish Borders, TD1 1QH, United Kingdom; Yale University School of Medicine, Pediatric Endocrinology & Diabetes, New Haven, Connecticut 06520-8064, United States

**Keywords:** X-linked hypophosphataemia (XLH), real-world data, disease monitoring program, registry, diagnosis

## Abstract

X-linked hypophosphatemia (XLH) is a phosphate-wasting disorder mediated by increased fibroblast growth factor 23 (FGF23) activity. Typical clinical features are skeletal deformities, muscle weakness, stiffness, and impaired physical function. Using real-world data from the XLH Disease Monitoring Program (XLH-DMP) and the International XLH Registry, this study sought to determine whether the age at which XLH is diagnosed differs between children with and without a family history of the disease. In both real-world studies, children with a family history of XLH were diagnosed at a younger age than those without a family history (XLH-DMP [n = 347]: mean age at diagnosis 1.6 [standard error (SE) 0.2] vs 2.7 [SE 0.2] years [*p* < .001]; International XLH Registry [n = 360]: mean age at diagnosis 1.8 [SE 0.2] vs 4.1 [SE 0.3] years [*p* < .001]). After controlling for sex, race, ethnicity, and country of residence (Cox proportional hazards model), children with a family history of XLH received a diagnosis of XLH at a younger age than those without a family history (XLH-DMP: hazard ratio 1.69, 95% confidence interval [CI] 1.33-2.16; International XLH Registry: hazard ratio 2.47, 95% CI 1.88-3.24). This study demonstrates that children without a family history of XLH are diagnosed at a significantly older age than those from families known to be affected, and that diagnosis may also be delayed despite a family history of XLH. A greater awareness of XLH and its early symptoms among pediatric healthcare professionals is required to avoid delays in diagnosis and treatment initiation.

## Graphical Abstract

**Graphical Abstract:**
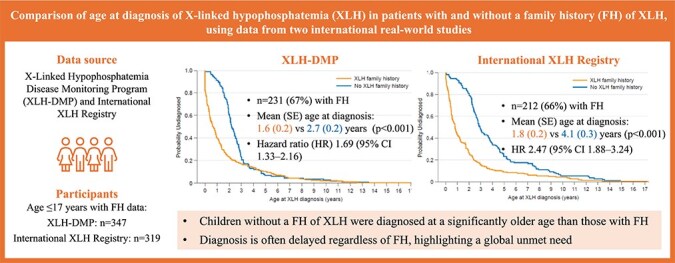

## Introduction

X-linked hypophosphatemia (XLH) is a rare, progressive phosphate-wasting disorder that is caused by loss-of-function variants in the *PHEX* (phosphate-regulating endopeptidase homologue, X-linked) gene. The resulting excess circulating levels of fibroblast growth factor 23 (FGF23) lead to renal phosphate wasting and hypophosphatemia.^1^^,^^2^ Chronic hypophosphatemia has deleterious effects on bone growth and quality, causes abnormalities in muscle function, and impairs dental mineralization.^1^^,^^3–5^

Clinical symptoms of XLH develop during the first or second year of life, although rachitic skeletal deformities may become apparent as early as 6 mo of age; children frequently present with delayed walking, a waddling gait, and progressive lower limb deformities during the second year of life.^6^ XLH in childhood is also associated with osteomalacia, short stature, and dental complications.^1^^–^^3^^,^^5–9^ Hypophosphatemia persists into adulthood, with continued osteomalacia and the accumulation of further skeletal morbidities, including fractures, pseudofractures, osteoarthritis with osteophytes, enthesopathy, and spinal stenosis.^4^^,^^6^^,^^10^^,^^11^ As a result of these complications, people with XLH experience pain, stiffness, and impaired physical function.^12^ People with XLH frequently require corrective surgery for skeletal deformities, hip and knee arthroplasty, spinal surgery, and surgical fixation of fractures.^10^^,^^11^

Delayed diagnosis, misdiagnosis, and delayed treatment in people with XLH are well documented^6^ and may lead to worse health outcomes and lifelong consequences.^2^^,^^13^^,^^14^ Thus, early diagnosis is likely to lead to earlier intervention and improved health outcomes.

XLH is transmitted in an X-linked dominant manner^15^^,^^16^ and various studies have reported that most people with XLH have a family history of the disease^17^^,^^18^; however, 20%-30% of people with XLH have spontaneous disease with no known family history.^17^^,^^19^^,^^20^ It might be expected that those with a known family history of XLH would be diagnosed earlier than those without a known family history. This is explored in the current study, using two prospective real-world datasets.

The XLH Disease Monitoring Program (XLH-DMP; NCT03651505) is an international, prospective, 10-yr, longitudinal, observational study of adults and children with XLH, which began in 2018 and has sites in North and South America.^21^^,^^22^ DMPs provide an alternative to traditional registries and extensive post-marketing studies, enabling enhanced data collection and collaboration between healthcare manufacturers, healthcare professionals, and patients.^23^ The International XLH Registry (NCT03193476) is an observational real-world data collection program that began in 2017 with sites across Europe and Israel.^24^

The objectives of the current analysis were to determine whether age at diagnosis differs between children with and without a family history of XLH, and to identify the potential influence of other factors such as sex, race, and country of residence.

## Materials and methods

### Data sources

The current analysis used data relating to the presence or absence of a family history of XLH from two sets of children (<18 yr of age at enrollment) as follows:

- Data captured up to 28 February 2023 from children who enrolled in the XLH-DMP between 2018 and 2022.
- Data at the first database lock (29 March 2021) from children who were enrolled in the International XLH Registry between 2017 and November 2020.^24^

The XLH-DMP has been collecting demographic, biochemical, clinical, disease severity, patient-reported outcomes, treatment, and disease progression data since 2018. Enrollment closed in December 2022. The population comprises adults and children with XLH in the USA, Argentina, Brazil, Canada, Chile, and Colombia. To be included in the XLH-DMP, individuals must have a clinical diagnosis of XLH based on one of: family history, confirmed *PHEX* variant, and/or a biochemical profile consistent with XLH. Key inclusion criteria are summarized in Table S3.

The International XLH Registry enrolled people with XLH in Europe and Israel.^24^^,^^25^ The International XLH Registry includes people of any age who, in the opinion of the treating physician, have a clinical presentation or radiological, biochemical, genetic, or family mapping investigation result that supported a diagnosis of XLH at the enrollment visit. *PHEX* variant analysis was not available for all participants, but those who had undergone such testing and were not found to carry a pathogenic or likely pathogenic *PHEX* variant were ineligible for inclusion in the registry. Key inclusion criteria are summarized in Table S3.

For both studies, people with XLH were enrolled regardless of current or prior treatment received for XLH (naïve to treatment, treated with phosphate and/or active vitamin D supplementation or burosumab, or continuing untreated); however, those who had been involved in an interventional clinical trial were not eligible until completion of that trial. Participants may have received treatment with burosumab through clinical prescription but treatment was not provided via either the XLH-DMP or the International XLH Registry.

### Definition of family history

The presence of a family history of XLH was defined as having a report of at least one biological parent affected by the condition. Neither study collected information about the timing of diagnosis for siblings (or other family members besides parents), diagnosis could have occurred before or after the study participant’s diagnosis; therefore, affected siblings could not be included in the categorization of presence or absence of family history. For the XLH-DMP the presence or absence of a family history of XLH was reported by the individual or their caregiver, for the International XLH Registry, family history was based on the person’s medical history.

### Ethics

Both the XLH-DMP and International XLH Registry are administered in accordance with the recommendations guiding physicians in biomedical research involving human subjects that were adopted in 1964 by the 18th World Medical Assembly, in Helsinki, Finland, with later revisions. Parental informed consent for inclusion in the XLH-DMP or International XLH registry was obtained from the child’s legally designated representative in line with national guidance. Assent was also sought from children of applicable age in line with national guidance.

### Analyses

Analysis was conducted on each dataset (XLH-DMP and International XLH Registry) separately because variables did not fully align across studies and diagnostic procedures differed, including the availability of genetic and FGF23 testing. However, sample sizes were considered sufficient to detect differences at the regional level (Americas vs Europe/Israel).

Patient characteristics (XLH family history, age at enrollment, sex, race, ethnicity, country of residence) were summarized by number of subjects, mean, and standard deviation (SD) for continuous variables, and by number and percentage of patients for categorical variables. Patients were also categorized according to their age at enrollment. The age categories (<1, 1 to <5, 5 to <12, and 12 to <18 yr) were selected to reflect those used in burosumab clinical trials and previous International XLH Registry publications.^18^^,^^26^^,^^27^ Details of the categories used in the analysis are provided in Table S4.

Age at diagnosis for those with and without a family history of XLH was compared for all patients and stratified by age categories. The F-test was used to ascertain equal or unequal variance, followed by comparison using the Student’s t-test for equal variance or Welch t-test for unequal variance.

Cox proportional hazards models were used to analyze associations between patient characteristics and age at XLH diagnosis, adjusting for age (continuous), sex, race, ethnicity (XLH-DMP only), country, and XLH family history. A generalized linear model (GLM) was used to predict age at XLH diagnosis using the same covariates as the Cox proportional hazards model. Kaplan-Meier analysis was used to determine the probability of XLH diagnosis by age at diagnosis in those with and without a family history of XLH.

There was no imputation of missing data apart from missing dates. Missing days were imputed as first of the month and missing months as January; missing years were not imputed.

## Results

### Patient populations

#### XLH-DMP

Data were available from 347 children with XLH in the XLH-DMP; all had family history data documented and were included in the analysis. Of these, 213 (61.4%) were female and 229 (66.0%) were from the USA. Two-thirds (231 [66.6%]) had a known family history of XLH. None of the children categorized as having no known family history of XLH had an identified affected sibling, compared with 48.9% of those with a known family history (Table 1).

**Table 1:** Comparison of patient characteristics at enrollment for children with and without a family history of XLH.

Characteristic		All children with XLH	Family history	No family history
**XLH-DMP**				
**N**		347	231	116
**Age (years) at enrollment, mean (SD)**		9.0 (4.8)	8.4 (4.9)	10.2 (4.4)
**Age at enrollment, *n* (%)**	<1 yr	8 (2.3)	8 (3.5)	0 (0.0)
	1 to <5 yr	80 (23.1)	63 (27.3)	17 (14.7)
	5 to <12 yr	154 (44.4)	101 (43.7)	53 (45.7)
	12 to <18 yr	105 (30.3)	59 (25.5)	46 (39.7)
**Sex, *n* (%)**	Female	213 (61.4)	128 (55.4)	85 (73.3)
	Male	134 (38.6)	103 (44.6)	31 (26.7)
**Sibling with XLH, *n* (%)**		113 (32.6)	113 (48.9)	0 (0.0)
**Race, *n* (%)**	White	254 (73.2)	177 (76.6)	77 (66.4)
	Non-white	45 (13.0)	23 (10.0)	22 (19.0)
	Unknown	48 (13.8)	31 (13.4)	17 (14.7)
**Ethnicity, *n* (%)**	Hispanic or Latino	103 (29.7)	62 (26.8)	41 (35.3)
	Not Hispanic or Latino	205 (59.1)	142 (61.5)	63 (54.3)
	Others	39 (11.2)	27 (11.7)	12 (10.3)
**Country, *n* (%)**	Argentina	30 (8.6)	16 (6.9)	14 (12.1)
	Brazil	24 (6.9)	17 (7.4)	7 (6.0)
	Canada	41 (11.8)	30 (13.0)	11 (9.5)
	Chile	16 (4.6)	12 (5.2)	4 (3.4)
	Colombia	7 (2.0)	3 (1.3)	4 (3.4)
	USA	229 (66.0)	153 (66.2)	76 (65.5)
**International XLH Registry**				
**N**		319	212	107
**Age (years) at enrollment, mean (SD)**		9.4 (4.5)	9.1 (4.7)	10.1 (4.1)
**Age at enrollment, *n* (%)**	<1 yr	5 (1.6)	4 (1.9)	1 (0.9)
	1 to <5 yr	64 (20.1)	51 (24.1)	13 (12.1)
	5 to <12 yr	143 (44.8)	88 (41.5)	55 (51.4)
	12 to <18 yr	107 (33.5)	69 (32.5)	38 (35.5)
**Sex, *n* (%)**	Female	190 (59.6)	121 (57.1)	69 (64.5)
	Male	129 (40.4)	91 (42.9)	38 (35.5)
**Sibling with XLH, *n* (%)**		90 (28.2)	86 (40.6)	4 (3.7)
**Race, *n* (%)**	White	179 (56.5)	113 (53.3)	66 (62.9)
	Non-white	22 (6.9)	10 (4.7)	12 (11.4)
	Unknown/not reported^a^	116 (36.6)	89 (42.0)	27 (25.7)
**Country, *n* (%)**	France	97 (30.4)	72 (34.0)	25 (23.4)
	Germany	31 (9.7)	20 (9.4)	11 (10.3)
	Italy	20 (6.3)	10 (4.7)	10 (9.3)
	Netherlands	18 (5.6)	9 (4.2)	9 (8.4)
	Spain	19 (6.0)	9 (4.2)	10 (9.3)
	UK	120 (37.6)	81 (38.2)	39 (36.4)
	Other	14 (4.4)	11 (5.2)	3 (2.8)
**Diagnosis confirmed by *PHEX* variant test, *n* (%)** ^b^		217 (68.0)	135 (63.7)	82 (76.6)

Abbreviations: *PHEX*, phosphate-regulating endopeptidase homologue, X-linked gene; SD, standard deviation; XLH, X-linked hypophosphatemia; XLH-DMP, XLH Disease Monitoring Program.

a Collection of race/ethnicity data is not permitted by law in France.

b *PHEX* variant data not available for XLH-DMP.

#### International XLH registry

Data were available from 360 children with XLH in the International XLH Registry; 319 had family history data documented and were included in the analysis; 190 (59.6%) were female, 120 (37.6%) were from the UK, and 97 (30.4%) from France. Two-thirds (212 [66.5%]) had a known family history of XLH; of those categorized as having no known family history of XLH, 4 (3.7%) had an identified affected sibling, compared with 40.6% of those with a known family history (Table 1).

### Age at diagnosis

#### XLH-DMP

**Figure 1:**
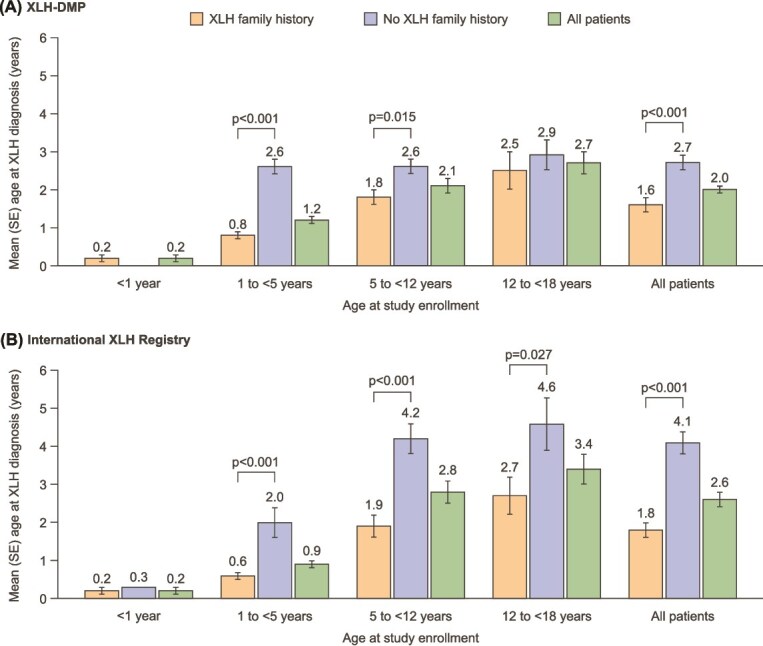
Age at diagnosis by XLH family history. Bars and numbers denote mean age (years), error bars denote standard error (SE). SE is not available for the international XLH registry category of “No XLH family history” for age at study enrollment <1 yr because this data was from one patient. Abbreviations: XLH, X-linked hypophosphatemia; XLH-DMP, XLH disease monitoring program.

Children with a family history of XLH were diagnosed at a significantly younger age than those without a family history (mean age at diagnosis 1.6 [standard error (SE) 0.2] vs 2.7 [SE 0.2] years, *p* < .001) (Figure 1A). This was the case for all subgroups analyzed based on age at enrollment in the XLH-DMP, although the difference was not significant for those aged 12 to <18 yr. There were no children aged <1 yr at enrollment without a family history of XLH.

**Figure 2:**
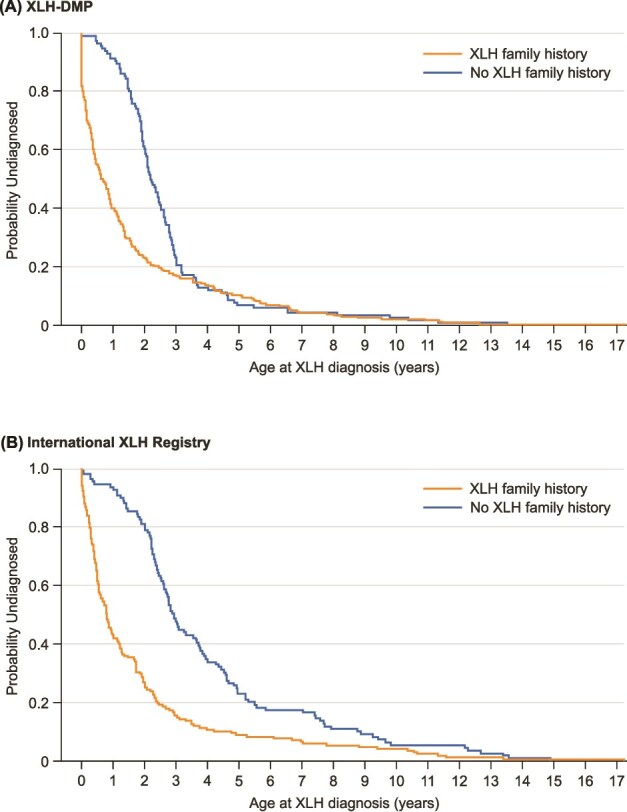
Kaplan-Meier plots for age at diagnosis in children with and without a family history of XLH. Kaplan-Meier rank tests for homogeneity: (A) XLH-DMP, log rank chi square 19.61 (*p* < .001); Wilcoxon chi square 61.71 (*p* < .001); (B) international XLH registry: log rank chi square 39.09 (*p* < .001); Wilcoxon chi square 69.34 (*p* < .001). Abbreviations: XLH, X-linked hypophosphatemia; XLH-DMP, XLH disease monitoring program.

In the Cox proportional hazards model, after controlling for sex, race, ethnicity, and country of residence, children with a family history of XLH were diagnosed with XLH at a younger age than those without a family history (hazard ratio 1.69; 95% confidence interval [CI] 1.33-2.16) (Table 2**,**  Figure 2A). Children who were older at enrollment (*p* < .001) or from Colombia (vs USA) (*p* = .005) were diagnosed at an older age. In the overall population, those who were Hispanic or Latino were diagnosed at a younger age (*p* = .029). The GLM analysis supported the results of the Cox proportional hazard model: children with a family history of XLH were more likely to be diagnosed at a younger age (estimate 0.10 [SE 0.03]; *p* < .01) (Table S5 and S6).

**Table 2:** Relationship between age at diagnosis and patient characteristics: cox proportional hazards model outcomes.

Parameter	Reference	Category	Hazard ratio	95% CI	Chi square	*p* value
**XLH-DMP**						
**XLH family history**	No	Yes	1.69	1.33, 2.16	17.94	**<0.001**
**Age at enrollment**			0.95	0.92, 0.97	18.68	**<0.001**
**Sex**	Male	Female	1.10	0.88, 1.38	0.68	0.410
**Race**	White	Non-White	0.95	0.65, 1.39	0.07	0.795
		Unknown/not reported	0.99	0.47, 2.08	0.00	0.975
**Ethnicity**	Not Hispanic or Latino	Hispanic or Latino	1.58	1.05, 2.38	4.77	**0.029**
		Others	2.02	0.87, 4.69	2.66	0.103
**Country**	USA	Argentina	0.72	0.42, 1.22	1.49	0.222
		Brazil	0.70	0.4, 1.23	1.53	0.216
		Canada	0.94	0.63, 1.39	0.10	0.751
		Chile	0.64	0.34, 1.20	1.91	0.167
		Colombia	0.28	0.12, 0.69	7.78	**0.005**
**International XLH Registry**						
**XLH family history**	No	Yes	2.47	1.88, 3.24	42.60	**<0.001**
**Age at enrollment**			0.88	0.85, 0.91	58.39	**<0.001**
**Sex**	Male	Female	0.93	0.72, 1.21	0.28	0.597
**Race**	White	Non-White	1.39	0.85, 2.26	1.70	0.192
		Unknown/not reported	0.52	0.29, 0.94	4.70	**0.030**
**Country**	UK	Germany	0.98	0.64, 1.50	0.01	0.916
		France	2.96	1.60, 5.46	12.03	**<0.001**
		Netherlands	0.73	0.40, 1.33	1.06	0.304
		Italy	1.32	0.83, 2.11	1.36	0.244
		Spain	0.96	0.57, 1.61	0.03	0.866
		Other	0.84	0.44, 1.59	0.30	0.585

Significant results (*p* < .05) shown in **bold**.

Ethnicity was not included in the International XLH Registry analysis because of substantial missing data (31%) and imbalance between groups (3% Hispanic/Latino, 58% not Hispanic/Latino, 9% unknown).

Abbreviations: CI, confidence interval; XLH, X-linked hypophosphatemia; XLH-DMP, XLH Disease Monitoring Program.

Kaplan-Meier analysis confirmed that children with a family history of XLH were diagnosed earlier (Figure 2A).

### International XLH registry

Children with a family history of XLH were diagnosed at a younger age than those without a family history (mean age at diagnosis 1.8 [SE 0.2] vs 4.1 [SE 0.3] years, *p* < .001) (Figure 1B). This was the case for all age subgroups based on age at enrollment, although the difference was not significant for those aged <1 yr at enrollment (this age group included only 5 children with a family history of XLH and 1 with no family history).

In the Cox proportional hazards model, after controlling for sex, race, ethnicity, and country of residence, children with a family history of XLH were diagnosed with XLH at a younger age than those without a family history (hazard ratio 2.47; 95% CI 1.88-3.24) (Table 2**,**  Figure 2B). Children who were older at enrollment (*p* < .001) were diagnosed at an older age than those enrolled at a younger age; those whose race was unknown/not reported (vs White) (*p* = .030) or from France (vs UK) (*p* < .001) were diagnosed at a younger age. The GLM analysis supported the results of the Cox proportional hazards model: children with a family history of XLH were more likely to be diagnosed at a younger age (estimate 0.23 [SE 0.04]; *p* < .001) (Table S5, Table S6).

Kaplan-Meier analysis confirmed that children with a family history of XLH were diagnosed earlier (Figure 2B).

## Discussion

This analysis of real-world data from more than 650 children (aged <18 yr) with XLH in the Americas, Europe, and Israel shows that those with a family history were diagnosed with XLH at an earlier age than those without a family history of the condition. This was seen in both the XLH-DMP and International XLH Registry and was maintained when controlling for age at study enrollment, sex, race, ethnicity, and country of residence. Age at study enrollment, race, ethnicity, and country of residence were also significantly associated with age at diagnosis.

These real-world data cover a broad demographic and provide a substantial sample size for analysis in the context of XLH as a rare disease. Whilst a pooled analysis may have been preferable, this was not possible because of differences between the two data sources in variables (eg, ethnicity, countries) and diagnostic procedures. However, the parallel analyses allow comparison of outcomes in the two populations.

Children with a family history of XLH were diagnosed at a similar mean age in the two studies (1.6 yr in the XLH-DMP; 1.8 yr in the International XLH Registry). However, it was also apparent that, even with a family history of XLH, diagnosis often occurred after the first years of life. Those aged 12 to <18 yr at study enrollment who had a family history of XLH had been diagnosed at a mean age of 2.5 yr in the XLH-DMP and 2.7 yr in the International XLH Registry. According to both the XLH-DMP and the International XLH Registry analysis, the probability of being undiagnosed by the age of 1 yr in those with a family history of XLH was 40%-42%. A positive family history would ideally prompt an early and thorough investigation in the first weeks or months of life for all infants born to women with XLH and the daughters of men with XLH.

The mean age at diagnosis for those without a family history of XLH differed substantially between the two studies: 2.7 yr in the XLH-DMP and 4.1 yr in the International XLH Registry. This difference in the age at diagnosis between the two studies may reflect differences in healthcare systems. In the UK and some other European countries, initial pediatric care is led by general practitioners or family physicians, with variable degrees of concentrated training in pediatrics,^28^ which could lead to delays in recognition, diagnosis, and referral to specialist pediatricians. Other potential reasons for regional differences could include variation in routine assessment of biochemical profiles, cultural attitudes to accepting/denying certain features of the disease as abnormal, and variable access to healthcare (including specialists and screening procedures). According to both the XLH-DMP and the International XLH Registry analyses, the probability of being undiagnosed at 1 yr of age in those without a family history of XLH was greater than 90%.

Delays in diagnosis may subsequently delay treatment. In XLH, there is evidence that delaying treatment with phosphate supplements and/or active vitamin D is associated with worse health outcomes such as slower linear growth, reduced final height, reduced bone mass accrual, greater bone deformities, and worse dental health.^6^^,^^14^^,^^29–32^ Delays in the diagnosis of rare diseases can also have economic implications for individuals and healthcare systems.^33^^,^^34^ Currently, evidence comparing the long-term impact of early treatment with burosumab (versus either no treatment or conventional therapy with active vitamin D and phosphate supplementation) is lacking for most outcomes including, for example, final height, limb deformities, and need for orthopedic surgery.

Increasing awareness of the presenting symptoms of XLH among healthcare professionals in primary care and across a range of secondary care specialties is key to earlier diagnosis in children both with and without a family history of XLH. These symptoms include rachitic skeletal deformities, delayed walking, a waddling gait, muscle weakness, and progressive lower limb deformities.^2^ Seefried and colleagues provide an overview of the clinical manifestations that present throughout the lifespan of people with XLH.^35^ Findings that may be present in infants (up to 1 yr of age) include craniosynostosis, frontal bossing, rickets, Chiari malformation, lower limb abnormalities, muscle weakness, and hypophosphatemia. These manifestations may also be present in older children (1-12 yr). The severity of symptoms may vary with age at presentatio.^35^ Children with XLH often present with rickets or genu varum; this should not be assumed to result from nutritional deficiencies, and the underlying causes should be explored. A presentation of rickets should prompt investigation for other signs of XLH, which could reduce delays in diagnosis^31^ and potentially prevent lifelong sequelae. Healthcare professionals need to be sensitive to subtle dysplasia-type features in infants, including frontal bossing, craniosynostosis, dolichocephaly, growth failure, and a disproportionate sitting to total height ratio. In countries without access to *PHEX* testing, if clinical signs are subtle or biochemistry is not yet conclusive (with or without a family history), infants should be monitored until the absence of the XLH phenotype can be confirmed.

Strategies to prevent delayed diagnosis in familial cases of XLH need to be implemented. Even without genetic testing, children whose parents have XLH should ideally undergo targeted biochemical testing before 6 mo of age to facilitate early diagnosis. A consensus statement from 27 clinical experts and endorsed by multiple European medical societies recommends that testing for *PHEX* variants is performed routinely in newborn babies of families affected by XLH.^2^ Targeted genetic testing can be performed rapidly and inexpensively when the pathogenic variant in the relative is already known.^2^ Prenatal screening may also be an option—some children included in the current analysis had XLH diagnosed in utero*.* Routine testing of all neonates is unlikely to be feasible. However, the Generation Study being conducted by Genomics England and NHS England is currently testing newborns for over 200 rare genetic conditions, including those without recognized signs.^36^ This study aims to screen up to 100 000 newborns, which could lead to early diagnosis and treatment of rare genetic disorders. Routine testing of phosphate levels at birth might prove more cost-effective than routine genetic testing,^31^ although phosphate levels may be normal in newborns with XLH.^37^ This phenomenon places even greater emphasis on the need for routine *PHEX* testing of infants with a positive family history of XLH. When diagnosing XLH, it is critical that serum phosphate concentrations are interpreted relative to age-matched normal values, given the progressive decline in serum phosphate concentrations with age.

Another critical diagnostic nuance is that while parathyroid hormone (PTH) levels are usually normal at diagnosis in XLH, there are exceptions, and multiple authors have reported secondary hyperparathyroidism in XLH even before treatment.^38^^,^^39^ This is important because hypophosphatemia may be attributed to the influence of PTH and XLH overlooked as a diagnostic consideration. Low circulating levels of 25-hydroxyvitamin D in XLH may also complicate the diagnosis, especially given the high prevalence of vitamin D deficiency worldwide.^40^

We propose that the following five-step diagnostic paradigm is implemented as far as possible: 1. explore the family history of affected individuals, including parents and siblings who may not be aware that they have XLH; 2. evaluate the infant of affected parents for subtle signs of rickets or skeletal dysplasia; 3. conduct biochemical testing (eg, serum and urine phosphate, alkaline phosphatase, PTH, FGF23, calcium, tubular phosphate reabsorption), including repeat testing if serum phosphate is initially normal but an index of suspicion for XLH persists; 4. undertake *PHEX* testing where clinical suspicion is high and testing is available; 5. educate family members about the potential for other family members to be affected and the importance of early diagnosis and treatment.

This study has several limitations. Real-world data can be limited by quality and validity. Family history status and age at diagnosis were reported by parents or caregivers in the XLH-DMP and may therefore be subject to recall bias. Defining family history based on affected parents alone could have overlooked participants with an affected older sibling. However, only 4 out of 666 children (0.6%) in the entire study (all from the International XLH Registry) without a parental family history of XLH had an affected sibling. Since it is not known if that affected sibling was older or younger, these 4 could not be reclassified reliably. In addition, the age at diagnosis was missing for some participants in the International XLH Registry, so those children were excluded from the analysis. The potential for selection bias was not addressed through this analysis (eg, disease severity, access to quality care, variation in care delivery across countries) and there may be residual confounding factors despite controlling for other characteristics. This study did not capture reported age at diagnosis in adults; XLH is sometimes diagnosed in adults after the condition is diagnosed in their children. People with XLH who have not been diagnosed clinically will not have been included in either dataset; therefore, the actual delay in diagnosis could be even greater for the overall XLH population. The legal restriction on reporting of race in some countries (eg, France) meant that this characteristic was “unknown” for many children, limiting the value of including race in the analysis.

Future research should evaluate whether diagnosis of XLH at an earlier age is associated with a corresponding reduction in the time to treatment initiation and improvements in clinical outcomes. An investigation into the age at which hypophosphatemia occurs in children with XLH is warranted. It would also be valuable to explore variability in the course and severity of the clinical manifestations of XLH, given the phenotypic spectrum of the disease.^35^^,^^41^^,^^42^

## Conclusions

Data from two real-world studies demonstrate that children without a family history of XLH were diagnosed at a significantly older age than those from families known to be affected. However, diagnosis was also frequently delayed in children with a family history of XLH. Thus, interventions may be delayed in people with XLH regardless of whether they have a family history of the condition. A greater awareness of XLH and its early symptoms among pediatric healthcare professionals is required to avoid delays in diagnosis and ensure timely treatment.

## Supplementary Material

XLH_age_at_diagnosis_manuscript_supplementary_materials_ziaf158

## Data Availability

Data that underlie the results reported in this article may be requested. Kyowa Kirin and Ultragenyx Pharmaceutical will review requests individually to determine whether requests are legitimate, relevant and meet sound scientific principles, and are within the scope of the participants’ informed consent.
