# Supplementary material for: Delayed diagnosis of X-linked hypophosphatemia in the absence of family history: a global unmet need
Source: JBMR Plus. 2025 Oct 7;9(12):ziaf158. doi: 10.1093/jbmrpl/ziaf158 (PMC12579927; doi:10.1093/jbmrpl/ziaf158)
Supplement: XLH_age_at_diagnosis_manuscript_supplementary_materials_ziaf158 [file xlh_age_at_diagnosis_manuscript_supplementary_materials_ziaf158.docx]

Supplementary material

Table S1 XLH-DMP Investigator Group Information

| Country | Last name | First name | Affiliation |
| --- | --- | --- | --- |
| **Argentina** | Arcari | Andrea | Hospital de Niños Ricardo Gutierrez |
|  | Brunetto | Oscar | Hospital Pedro de Elizalde |
|  | Douthat | Walter Guillermo | Hospital Privado Universitario de Córdoba |
| **Brazil** | Matsunaga Martin | Regina | Hospital das Clinicas da Universidade de São Paulo (HCFMUSP) |
|  | Moreira | Carolina A. | Internal Medicine and Endocrine Division (SEMPR) of Federal University of Parana, Academic Research Center of Pro-Renal Institute |
| **Canada** | Basak | Sanjukta | British Columbia Children's Hospital |
|  | Glorieux | Francis | Shriners Hospitals for Children |
|  | Khan | Sarah | Bone Research and Education Clinic and Credit Valley Hospital |
|  | Ward | Leanne | Children’s Hospital of Eastern Ontario, University of Ottawa |
| **Chile** | Florenzano | Pablo | Pontificia Universidad Católica de Chile |
| **Colombia** | Baquero Rodriguez | Richard | Hospital Universitario San Vicente Fundación, Universidad de Antioquia |
|  | Meza-Martinez | Adriana | Hospital Infantil Universitario de San José |
| **US** | Ashraf | Ambika P. | University of Alabama at Birmingham |
|  | Bowden | Sasigarn | Nationwide Children's Hospital |
|  | Bradley | Dixon P. | University of Colorado School of Medicine |
|  | Carpenter | Thomas | Yale University |
|  | Crane | Janet | The Johns Hopkins University School of Medicine |
|  | Dahir | Kathryn M. | Vanderbilt University Medical Center |
|  | Glass | Ian | Seattle Children's Hospital |
|  | Gottesman | Gary | Washington University School of Medicine |
|  | Gyuricsko | Eric | Eastern Virginia Medical School at Macon & Joan Brock Virginia Health Sciences Old Dominion University |
|  | Holm | Ingrid | Boston Children’s Hospital |
|  | Imel | Erik | Indiana University School of Medicine |
|  | Ing | Steven | The Ohio State University Wexner Medical Center |
|  | Jan de Beur | Suzanne | University of Virginia |
|  | Levine | Michael | Children's Hospital of Philadelphia |
|  | Paloian | Neil | University of Wisconsin School of Medicine & Public Health |
|  | Portale | Anthony | University of California San Francisco Medical Center |
|  | Rodriguez-Buritica | David | The University of Texas Health Science Center at Houston |
|  | Ryabets-Lienhard | Anna | Children’s Hospital of Los Angeles, University of Southern California, Keck School of Medicine |
|  | Simmons | Jill | Vanderbilt University Medical Center |
|  | Singh | Puja | University of California San Diego, Rady Children's Hospital San Diego |
|  | Tabatabai | Laila | Houston Methodist Research Institute |
|  | Wasserman | Halley | Cincinnati Children's Hospital |
|  | Weber | Thomas | Duke University Medical Center |

Table S1 International XLH Steering Committee Group Information

| Country | Last name | First name | Affiliation |
| --- | --- | --- | --- |
| **Denmark** | Beck-Nielsen | Signe Sparre | Aarhus University Hospital, Aarhus |
| **Finland** | Mäkitie | Outi | University of Helsinki and Helsinki University Hospital, Helsinki |
| **France** | Briot | Karine | AP-HP, Cochin Hospital, Paris |
| **Germany** | Haffner | Dieter | Hannover Medical School, Hannover |
|  | Schnabel | Dirk | Charité, University Medicine, Berlin |
| **Israel** | Tripto-Shkolnik | Liana | Tel Aviv University, Tel Aviv, Israel and Sheba Medical Center, Tel HaShomer |
| **Italy** | Brandi | Maria Luisa | University Vita-Salute San Raffaele, Milan, Italy and FIRMO Foundation, Florence |
|  | Francesco | Emma | Bambino Gesù Children’s Hospital IRCCS, Rome |
|  | Giannini | Sandro | University of Padova, Padova |
| **Netherlands** | Levtchenko | Elena | Amsterdam University Medical Centre, Amsterdam |
|  | Zillikens | Carola M | Erasmus MC, University Medical Center Rotterdam, Rotterdam |
|  | Boot | Annemieke M | University Medical Center Groningen, University of Groningen, Groningen |
| **Spain** | Ariceta | Gema | University Hospital Vall d’Hebron, Autonomous University of Barcelona, Barcelona |
|  | de Lucas Collantes | Carmen | Hospital Infantil Universitario Niño Jesús and Universidad Autónoma de Madrid, Madrid |
| **Sweden** | Nilsson | Ola | Karolinska Institutet and University Hospital, Stockholm;  Örebro University and University Hospital, Örebro |
| **UK** | Keen | Richard | Royal National Orthopaedic Hospital, Stanmore |
|  | Padidela | Raja | Manchester University Hospital, Manchester, University NHS Foundation Trust, Manchester |

Table S3 Key study inclusion criteria for the XLH-DMP and International XLH Registry

| **Characteristic** | **XLH-DMP (NCT03651505)** | **International XLH Registry (NCT02915705)** |
| --- | --- | --- |
| **Age** | Any | Any |
| **Diagnosis** | Clinical diagnosis of XLH based on clinical features, including short stature or leg deformities *and* biochemical profile consistent with XLH, including documented history of hypophosphatemia, *or* confirmed *PHEX* variant in patient or in a family member | In the opinion of the treating physician, the patient has a clinical presentation, radiological, biochemical, genetic, or family mapping investigation result that supports the diagnosis of XLH |
| **Definition of family history of XLH** | Parent(s) affected by XLH | Parent(s) affected by XLH |
| **Prior treatment** | Untreated individuals, individuals treated with burosumab, or those treated with phosphate and active vitamin D metabolites/analogs, as prescribed by their physician | Individuals can be naïve to treatment, treated with conventional therapy (oral phosphate and/or active vitamin D analog), treated with burosumab, or currently untreated, as determined by their physician |
| **Clinical trial participation** | Individuals who are concurrently enrolled in a clinical trial are not eligible without prior approval from the Sponsor  All people with XLH who participated in burosumab clinical trials will be invited to participate | Individuals currently participating in an interventional clinical trial are excluded. People with XLH will be approached for inclusion into the International XLH Registry once their involvement in an interventional trial ends (including the completion of all trial follow-up assessments) |
| **Location** | USA, Canada, and Latin America | Europe and Israel |

Table S4 Categories explored in the analysis

| Characteristic | XLH-DMP | International XLH Registry |
| --- | --- | --- |
| Age at enrolment (years) | <1, 1 to <5, 5 to <12, 12 to <18 | |
| Sex | Male, female | |
| Race | White, non-white, unknown/not reported | |
| Ethnicity | Hispanic or Latino  Not Hispanic or Latino  Others | Not included because of substantial missing data and imbalance between groups (3% Hispanic/Latino, 58% not Hispanic/Latino, 9% unknown, 31% missing) |
| Country | Argentina, Brazil, Canada, Chile, Colombia, USA | France, Germany, Italy, Netherlands, Spain, UK, other |

Table S5 Relationship between age at diagnosis and patient characteristics: generalized linear model outcomes

| Parameter | Reference | | | Category | Estimate | | SE | | P value | |
| --- | --- | --- | --- | --- | --- | --- | --- | --- | --- | --- |
| XLH-DMP | | | | | | | | | | |
| Intercept (age at diagnosis, years) | | | | | 1.802 | | 0.419 | | **<0.001** | |
| XLH family history | No | Yes | | | −0.860 | | 0.274 | | **0.002** | |
| Age at enrollment | | | | | 0.100 | | 0.027 | | **0.002** | |
| Sex | Male | | | Female | −0.003 | | 0.262 | | 0.991 | |
| Race | White | | | Non-White | 0.477 | | 0.428 | | 0.267 | |
|  |  |  |  | Unknown/ not reported | 0.189 | | 0.875 | | 0.829 | |
| Ethnicity | Not Hispanic or Latino | | | Hispanic or Latino | −1.086 | | 0.466 | | **0.020** | |
|  |  |  |  | Others | −1.543 | | 0.990 | | 0.120 | |
| Country | USA | | | Argentina | 0.734 | | 0.620 | | 0.238 | |
|  |  |  |  | Brazil | 0.651 | | 0.641 | | 0.311 | |
|  |  |  |  | Canada | 0.564 | | 0.462 | | 0.222 | |
|  |  |  |  | Chile | 0.980 | | 0.716 | | 0.172 | |
|  |  |  |  | Colombia | 3.651 | | 1.006 | | **<0.001** | |
| International XLH Registry | | | | | | | | | | |
| Intercept (age at diagnosis, years) | | | | | | 2.058 | | 0.566 | | **<0.001** |
| XLH family history | No | | Yes | | | −2.057 | | 0.358 | | **<0.001** |
| Age at enrolment | | | | | | 0.233 | | 0.039 | | **<0.001** |
| Sex | Male | | Female | | | 0.166 | | 0.490 | | 0.624 |
| Race | White | | Non-white | | | −0.900 | | 0.642 | | 0.163 |
|  |  |  | Unknown/ not reported | | | 1.771 | | 0.773 | | **0.023** |
| Country | UK | | Germany | | | −0.479 | | 0.611 | | 0.434 |
|  |  |  | France | | | −2.915 | | 0.803 | | **<0.001** |
|  |  |  | Netherlands | | | 0.232 | | 0.793 | | 0.771 |
|  |  |  | Italy | | | −0.620 | | 0.682 | | 0.364 |
|  |  |  | Spain | | | −0.331 | | 0.720 | | 0.646 |
|  |  |  | Other | | | 0.721 | | 0.848 | | 0.397 |
| Ethnicity was not included in the International XLH Registry analysis because of substantial missing data (31%) and imbalance between groups (3% Hispanic/Latino, 58% not Hispanic/Latino, 9% unknown).  Significant results (p<0.05) shown in **bold.**  SE, standard error; XLH, X-linked hypophosphatemia | | | | | | | | | | |

Table S6 Fit statistics for the generalized linear model analysis

| Source | **DF** | **Sum of squares** | **Mean square** | **F value** | **P value** |
| --- | --- | --- | --- | --- | --- |
| XLH-DMP | | | | | |
| Model | 12 | 333.8 | 27.8 | 5.3 | <0.001 |
| Error | 334 | 1760.9 | 5.3 |  |  |
| Corrected total | 346 | 2094.7 |  |  |  |
| Age at enrollment | 1 | 72.3 | 72.3 | 13.7 | **<0.001** |
| Sex | 1 | 0.0 | 0.0 | 0.0 | 0.991 |
| Race | 2 | 6.5 | 3.3 | 0.6 | 0.539 |
| Ethnicity | 2 | 35.7 | 17.9 | 3.4 | **0.035** |
| Family history | 1 | 51.8 | 51.8 | 9.8 | **0.002** |
| Country | 5 | 79.2 | 15.8 | 3.0 | **0.012** |
| International XLH Registry | | | | | |
| Model | 1 | 687.6 | 62.5 | 9.0 | <0.001 |
| Error | 24 | 1710.2 | 6.9 |  |  |
| Corrected total | 25 | 2397.7 |  |  |  |
| Age at enrollment | 1 | 260.9 | 260.9 | 37.7 | **<0.001** |
| Sex | 1 | 1.7 | 1.7 | 0.3 | 0.624 |
| Race | 2 | 56.1 | 28.1 | 4.1 | **0.019** |
| XLH family history | 1 | 229.0 | 229.0 | 33.08 | **<0.001** |
| Country | 6 | 141.0 | 23.5 | 3.4 | **0.003** |
| Significant results are shown in **bold** (p<0.05)  DF, degrees of freedom; DMP, disease monitoring program; XLH, X-linked hypophosphatemia | | | | | |
